# Osteogenic Potential of a Polyethylene Glycol Hydrogel Functionalized with Poly-Lysine Dendrigrafts (DGL) for Bone Regeneration

**DOI:** 10.3390/ma16020862

**Published:** 2023-01-16

**Authors:** Sandra Roumani, Charlotte Jeanneau, Thomas Giraud, Aurélie Cotten, Marc Laucournet, Jérôme Sohier, Martine Pithioux, Imad About

**Affiliations:** 1 Aix-Marseille University, CNRS, ISM, 13009 Marseille, France; 2 APHM, Hôpital Timone, Pôle Odontologie, 13005 Marseille, France; 3 Laboratory for Tissue Biology and Therapeutic Engineering (LBTI), UMR 5305, CNRS, Lyon University, 69367 Lyon, France; 4 Aix-Marseille University, APHM, CNRS, ISM, Sainte-Marguerite Hospital, Institute for Locomotion, Department of Orthopaedics and Traumatology, 13009 Marseille, France

**Keywords:** poly-lysin dendrigraft, hydrogel, bone regeneration scaffold, stem cell chemotaxis, osteogenic potential

## Abstract

Resorbable hydrogels are widely used as scaffolds for tissue engineering. These hydrogels can be modified by grafting dendrimer-linked functionalized molecules (dendrigrafts). Our aim was to develop a tunable poly(L-lysine) dendrigrafts (DGL)/PEG-based hydrogel with an inverse porosity and to investigate its osteogenic potential. DGL/PEG hydrogels were emulsified in a surfactant-containing oil solution to form microspheres. The toxicity was evaluated on Human Vascular Endothelial Cells (HUVECs) and Bone Marrow Mesenchymal Stem Cells (hMSCs) with Live/Dead and MTT assays. The effects on HUVECs were investigated through C5 Complement expression by RT-PCR and C5a/TGF-β1 secretion by ELISA. Recruitment of hMSCs was investigated using Boyden chambers and their osteogenic differentiation was studied by measuring Alkaline Phosphatase activity (ALP) and BMP-2 secretion by ELISA. Adjusting the stirring speed during the emulsification allowed to obtain spherical microspheres with tunable diameters (10–1600 µm). The cell viability rate with the hydrogel was 95 and 100% with HUVECs and hMSCs, respectively. Incubating HUVECs with the biomaterial induced a 5-fold increase in TGF-β1 and a 3-fold increase in Complement C5a release. Furthermore, HUVEC supernatants obtained after incubation with the hydrogel induced a 2.5-fold increase in hMSC recruitment. The hydrogel induced a 3-fold increase both in hMSC ALP activity and BMP-2 secretion. Overall, the functionalized hydrogel enhanced the osteogenic potential by interacting with endothelial cells and hMSCs and represents a promising tool for bone tissue engineering.

## 1. Introduction

Bone regeneration is a well-orchestrated process which occurs through successive steps including hematoma formation, acute inflammatory reaction, granulation tissue formation, bone regeneration and remodeling [1,2,3,4,5]. During this process, mesenchymal stem cells (MSCs) are recruited from the bone marrow, the periosteum, and the endosteum, and differentiate towards the chondrogenic and osteogenic lineages to regenerate bone [5]. In small size defects, this process leads to a spontaneous bone regeneration. However, if a scaffold is not used in critical-sized defects, bone regeneration may be compromised [6,7]. Strategies for bone regeneration have been developed based on bone grafts and various substitutes including autologous, allogenic, and xenogenic bone grafts. While the autologous bone graft has been considered as the gold standard for critical size bone regeneration [8], major drawbacks have been reported including donor site morbidity, risk of infection, and reduced graft volume [7]. Current bone regeneration strategies are based on applying osteo-conductive or osteo-inductive scaffolds. In the osteo-inductive strategy, a scaffold containing/loaded with bioactive molecules such as growth factors is used. This allows the host progenitor cell recruitment, proliferation, and differentiation. In the osteo-conductive strategy, the scaffold structure/porosity allows its passive colonization by the host stem cells and ingrowth of blood vessels to regenerate the missing bone [9,10].

Resorbable materials in the form of granules, particles or microspheres have long been used as osteo-conductive scaffolds in critical size bone defects regeneration. These provide an appropriate environment for cell adhesion, migration, proliferation, and osteogenic differentiation. They also play a crucial role in supporting ingrowth of new blood vessels [11]. A variety of scaffold materials including ceramics, synthetic polymers, and composite materials are used in this strategy [12]. In addition, biomaterials prepared as injectable hydrogels or microspheres have drawn attention with their minimal invasiveness. These materials are easy to handle and can fill in the defect space and match irregular and complex defects [13]. Additionally, their use reduces the infection risk, scarring, and post-operative pain. Hydrogels form the major part of currently used tissue engineering scaffolds [13,14]. Based on their origin, hydrogels can be classified into three categories: naturally derived hydrogels (e.g., collagen, hyaluronic acid, gelatin, fibrin, etc.), synthetic hydrogels (e.g., poly(ethylene) glycol (PEG)), and semi-synthetic hydrogels (PEG combined with a cholesterol-bearing polysaccharide). In addition, when prepared in the form of microspheres, hydrogels provide an inverse porosity between the microspheres that can be colonized by host stem cells and vascularized by ingrowth of newly formed blood vessels [15,16].

Poly-L-lysine (PLL) of extracellular matrix proteins is a small natural homopolymer of the essential amino acid L-lysine frequently used to coat culture substrates. PLL functions as an attachment factor that enhances cell adhesion due to its strong affinity to proteins and electrostatic interactions between the positive charges on the PLL molecule and the negative charges on the cell membrane [17]. Thus, PLL is commonly used to enhance cell adhesion, spreading, proliferation and differentiation [18,19,20]. It has been shown that poly(ethylene terephthalate) stent surface modification with PLL promotes endothelial cell adhesion and growth [21,22]. In addition, several studies have shown that MSC adhesion, spread, proliferation and chondrogenic and osteogenic differentiation are enhanced in PLL-loaded hydrogels [23,24,25]. In particular, PLL-coated surfaces have been shown to upregulate genes involved in human bone marrow MSC (BMMSCs) adhesion, proliferation, and differentiation [26]. Poly(L-lysine) (PLL) dendrigrafts (DGL) are arborescent biosynthetic polymers of regular and controlled structures. When these dendritic structures with terminal amine groups are used for surface coating, they increase cell adhesion and proliferation as compared to PLL coating alone [27]. Synthesis of PEG hydrogels, containing dendritic or hyperbranched fragments, with very promising biomedical applications is not limited solely to the poly(L-lysine) dendrigrafts. Indeed, previous works demonstrated fabrication of hydrogels with hyperbranched molecules as well as their applications in the repair of corneal wounds and in orthopedic surgery for the treatment of osteoarthritis [28,29].

Recently, injectable poly(L-lysine) dendrigrafts (DGL)/PEG-based hydrogels with an inner porosity have been created using an effervescent reaction. Cell survival, adhesion, and proliferation allowed the formation of the new tissue inside the porous scaffold. Additionally, after a subcutaneous transplantation into mice, the hydrogel supported an extensive neo-vascularization [30].

The aim of this study was to prepare an injectable poly(L-lysine) dendrigraft (DGL) hydrogel in the form of tailorable microspheres with an inverse porosity and to investigate its osteogenic properties for potential use in bone regeneration [31]. We evaluated the hydrogel toxicity in endothelial and mesenchymal stem cells as both cell types play a major role in bone regeneration. Then, the hydrogel interaction with endothelial cells was studied by investigating the release of chemotactic factors such as TGF- β1 and Complement C5a. The effect of the hydrogel on mesenchymal stem cell osteogenic potential was investigated through their recruitment, BMP-2 secretion, and Alkaline phosphatase activity.

## 2. Materials and Methods

### 2.1. Reagents

Cells and culture media were obtained from PromoCell (Heidelberg, Germany). Culture materials and reagents were obtained from Dominique Dutscher (Brumath, France). ELISA kits were purchased from R&D Systems (Lille, France), Live/Dead^®^ staining kit from Thermo Fisher Scientific (Waltham, MA, USA). For the microspheres preparation, PEG-bis(N-succinimidyl succinate) (PEG-NHS), anhydrous dimethylsulfoxide (DMSO) and SPAN80^®^ were obtained from Sigma-Aldrich (St Louis, MO, USA), and poly(L-lysine) dendrigrafts (DGL) were obtained from COLCOM (Clapiers, France).

### 2.2. DGL/PEG Microspheres Preparation

**Figure 1:**
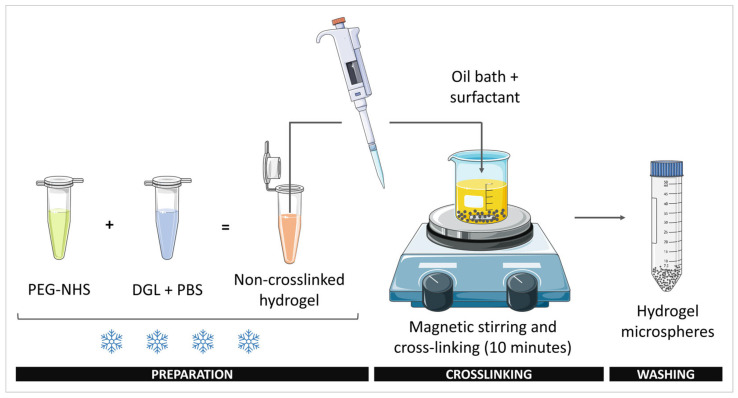
Representative sketch of the protocol used to prepare the DGL/PEG hydrogel microspheres. PEG-NHS was added to DGL prepared in PBS buffer on ice to inhibit the initiation of crosslinking. After vigorous homogenization on ice, the hydrogel is transferred to an oil bath containing the surfactant to allow crosslinking under magnetic stirring. After 10 min, the obtained microspheres are washed before use.

Poly(L-lysine) dendrigrafts (DGL, third generation, 22,000 g/mol) and PEG-bis(N-succinimidyl succinate) (PEG-NHS, 2000 g/mol) were first solubilized at 400 mg/mL in phosphate buffered saline (PBS) and anhydrous DMSO, respectively. Each stock solution was added to a determined volume of PBS in 2 mL tube followed by vigorous homogenization to obtain the desired concentrations of DGL and PEG in a fixed final volume. The hydrogel was prepared on ice to inhibit the initiation of cross-linking [20]. Once homogenized, the resulting mixed solution was injected in less than 5 s into an oil bath in a 50 mL beaker containing 20 mL of a mix of mineral oil and SPAN 80^®^ surfactant under agitation with a stirring bar. Stirring of the oil bath was pursued for 10 min to allow the hydrogel cross-linking and microsphere formation. Afterwards, the obtained microspheres were resuspended with manual stirring and ultrasound bath 3 times for 10 s, centrifuged for 10 min at 5000 rpm, washed 3 times with 20 mL of PBS until the solution became clear, and stored in PBS at 4 °C. For the in vitro assays, the hydrogel microspheres were sterilized overnight in EtOH:PBS (70:30, *v*/*v*) solution, washed 3 times for 30 min with 20 mL of sterile PBS, and kept at 4 °C prior to use (Figure 1).

### 2.3. Factorial Design and Characterization of DGL/PEG Microspheres

To investigate the effect of the processing parameters on DGL/PEG microsphere diameters, a full two-level factorial design (randomized 16 runs with three random center points) was performed. Four easily adjustable parameters were considered as variables in the factorial design: the hydrogel volume (50 to 200 µL), the stirring speed during water-in-oil emulsion (100 to 1300 rpm), the surfactant concentration (0.5 to 3 vol%) and the hydrogel composition (25 to 50 mg/mL of DGL for 50 mg/mL of PEG).

For each run, DGL/PEG microspheres average diameter was evaluated by image analysis using Image J open-source software (v.1.53t). Prior to the image acquisition with a light microscope (Leica, Wetzlar, Germany), 2 µL of an eosin alcoholic solution (Abcam, Cambridge, UK) was added to 50 µL of each microsphere batch, which was subsequently deposited on a glass slide and covered with a coverslip. For each sample, between 5 and 43 pictures were taken randomly at the same magnification. They were studied using the ‘analyze particle’ tool after thresholding of the pink microspheres. For each microsphere, the diameter was calculated from the measured area and 310 values were obtained on average for each sample. From these data, the average diameter was calculated, and variance analysis performed using statgraphics (Statpoint technologies, Inc., Warrenton, VA, USA).

### 2.4. Endothelial and Mesenchymal Stem Cell Culture

Human umbilical vein endothelial cells (HUVECs) were cultured in endothelial cell growth medium 2 (ECGM-2). These commercially available cells are derived from the endothelium of veins from the human umbilical cord. Human mesenchymal stem cells (hMSCs) from the bone marrow were cultured in hMSCs growth medium 2 (MGM-2). Cells were incubated at 37 °C, 5% CO_2_ atmosphere, cultured until reaching confluency, and sub-cultured. Culture medium was refreshed every 3 days.

### 2.5. Cell Treatment with the DGL/PEG Hydrogel

**Figure 2:**
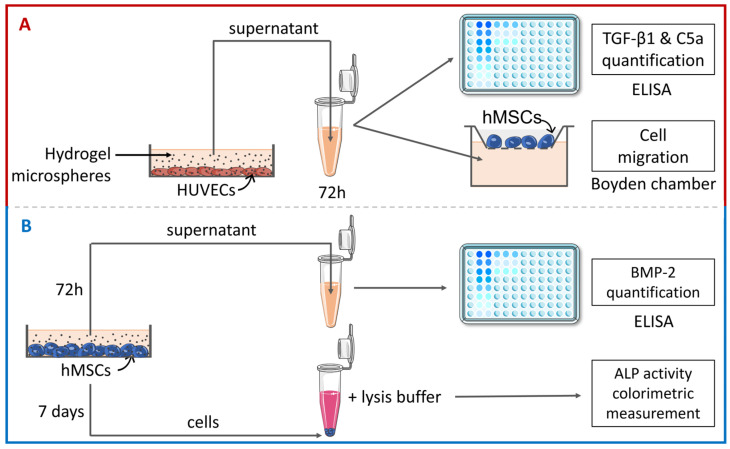
Representative sketch of the experimental protocol. (**A**) HUVECs and hMSCs were incubated with the hydrogel. After 72 h, the HUVEC supernatants were harvested and TGF-β1 and C5a secretion was quantified by ELISA. Migration of hMSCs was studied using Boyden chambers with HUVEC supernatants. (**B**) Secretion of BMP-2 was quantified after 72 h in hMSC supernatants by Elisa while hMSC differentiation was investigated after 7 days by measuring Alkaline Phosphatase (ALP) activity on cell lysates using a colorimetric method.

In order to prepare the hydrogel microspheres for the cell treatment, we used a mixture of 50 mg/mL of DGL with 50 mg/mL of PEG prepared in an oil bath containing 0.5% surfactant and stirred at 1300 rpm. HUVECs and hMSCs were seeded into 6-well plates and cultured in their respective media. At confluency, the hydrogel microspheres were added to the cells (25 microspheres/cm^2^). After 72 h, the cell viability was determined, and the supernatants were harvested to study cell migration and perform Elisa tests. ALP quantification was performed after 7 days directly on lysed cells (Figure 2A,B).

### 2.6. Cell Viability

Two methods were used to evaluate toxicity.

#### 2.6.1. Qualitative Method

In order to check the toxicity of the hydrogel, a Live/Dead^®^ assay (Thermo Fisher Scientific, L3224) was performed according to the manufacturer’s instructions. Briefly, after incubation with the hydrogel microspheres (25 microspheres/cm^2^) for 72 h, HUVECs and hMSCs were washed with PBS. We added a mix of Calcein AM and Ethidium Homodimer-1 (EthD-1) at 1 and 4 µM, respectively, prepared in 1 mL of PBS. After 30 min of incubation with the cells at 37 °C and 5% CO_2_, the cells were washed two times with PBS and observed with fluorescence equipped light microscope (Axio Observer A1, Carl Zeiss Microscopy, Jena, Germany) within 20 min (live cells excitation at 517 nm and emission at 494 nm; dead cell excitation at 617 nm and emission at 528 nm). A positive control was performed by incubating the cells with Triton™ 1% for 15 min at room temperature prior to staining.

#### 2.6.2. Quantitative Method

The quantitative evaluation of the cell viability was performed using the 3-(4,5-di-methylthiazol-2-yl)-2,5-diphenyltetrazolium bromide (MTT) test. After incubating the HUVECs and hMSCs for 72 h with the hydrogel microspheres (25 microspheres/cm^2^), the supernatants were removed, and immediately replaced with 1 mL/well of MTT solution (0.5 mg/mL) (Thermo Fisher Scientific, Waltham, MA, USA) for 2 h at 37 °C and 5% CO_2_. Supernatants were removed, and the produced crystals were solubilized with 1 mL/well of dehydrated DMSO. The absorbance was recorded at 550 nm with a microplate reader (Σ960; MeterTech, Taipei, Taiwan). Results were expressed as percent of controls (untreated cells).

### 2.7. C5 Complement Expression by Endothelial Cells

After incubating HUVECs for 72 h with the hydrogel (25 microspheres/cm^2^), the cells were harvested, and total RNAs were immediately isolated using a PureLink RNA minikit (Life Technologies, Oslo, Norway). RNA samples (2 µg) were reverse transcribed using a reverse transcription AMV system (Promega, Madison, WI, USA). Primers used were C5 forward 5′-AGTGTGTGGAAGGGTGGAAG-3′ and reverse 5′-GTTCTCTCGGGCTTCAACAG-3′; and Glyceraldehyde 3-phodphate dehydrogenase (GAPDH) as an internal control: forward 5′-GAAGGTGAAGTTCGGAGTC-3′ and reverse 5′-GAAGATGGTGATGGGATTTC-3′. PCR conditions were 94 °C for 2 min, then 30 cycles (94 °C for 30 s, 55 °C for 30 s, and 68 °C for 30 s), and 68 °C for 2 min. PCR products were separated onto 1.5% agarose gels.

### 2.8. Growth Factors and C5a Complement Protein Quantification

HUVECs and hMSCs were cultured with the hydrogel microspheres (25 microspheres/cm^2^) to investigate their effects on growth factor secretion. After incubation for 72 h, the supernatants were harvested to quantify TGF-β1 and C5a secretion by HUVECs and BMP-2 secretion by hMSCs using an enzyme-linked immunosorbent assay (ELISA) according to the manufacturer’s instructions (DuoSet ELISA Development System kit, R&D Systems, Lille, France).

### 2.9. Mesenchymal Stem Cell Migration

Migration of hMSCs was studied using Boyden chambers (8 µm pore size) in 24-well plates. hMSCs (10^5^ cells/100 µL) were seeded in the upper chamber while supernatants from HUVECs (+/− hydrogel) were placed in the lower chamber (500 µL per well). Serum-free hMSC culture medium was used as a control. After 24 h, cells migrating through the porous membrane were fixed (15 min, cold ethanol 70%) and stained with hematoxylin (20 min). The number of migrating cells was counted in 5 random fields using light microscopy. Results are expressed as number of migrating cells.

### 2.10. Mesenchymal Stem Cell ALP Activity

Osteogenic differentiation of hMSCs was studied by quantifying alkaline phosphatase (ALP) enzyme activity using a colorimetric ALP Kit (Abcam). hMSCs (10^5^ cells) seeded in 6-well plates were incubated (+/− 25 microspheres/cm^2^) for 7 days. Upon reaching confluency, the cells were dissociated using trypsin and counted. After rinsing, they were collected using 50 µL/10^5^ cells lysis buffer and three-time 10-s ultrasonic bath. Samples were centrifuged at 4 °C for 15 min to remove any insoluble material and the supernatant was collected and kept on ice. Standards and samples were placed in a 96-well plate and ALP measurement was performed according to the manufacturer’s instructions. Absorbance was measured at OD 405 nm with a microplate reader (Σ960, MeterTech, Taipei, Taiwan). Results are expressed as the percentage of control.

### 2.11. Statistical Analysis

All experiments were performed in triplicates with 3 different cell populations. Statistical significance was determined using the Student’s *t*-test to compare two sets of data from the different treatments and their respective controls. Data were expressed as means ± standard deviation and considered significant for *p* < 0.05.

## 3. Results

### 3.1. Production of Tunable Poly(L-Lysine) Dendrigrafts (DGL)/PEG-Based Hydrogel Microspheres

**Figure 3:**
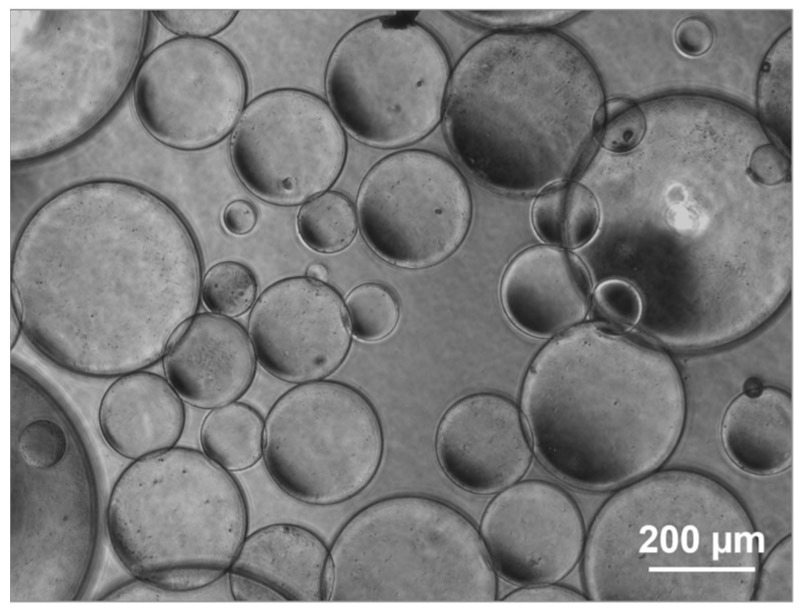
A representative image of DGL/PEG hydrogel microspheres. Illustration of the microsphere morphology obtained at a stirring speed of 1300 rpm. The spheres display different sizes, but the majority had a diameter of 200–300 µm as illustrated under optical microscopy. Scale bar: 200 µm.

A water-in-oil method was employed to prepare the hydrogel in the form of microspheres. The crosslinking reaction of DGL/PEG was very rapid and took place within seconds. This allowed to create an emulsion prior to the hydrogel formation, which occurred in each of the water-containing droplets within the oil phase. The resulting microspheres exhibited a spherical shape as assessed by optical microscopy (Figure 3).

**Figure 4:**
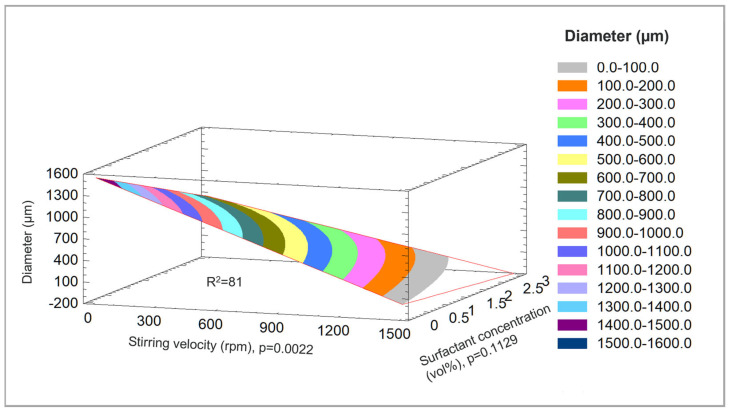
Full two-level factorial design of the DGL/PEG hydrogel microspheres preparation. Average microsphere diameters obtained in function of the stirring speed during water in oil emulsion, and surfactant concentration.

To control the DGL/PEG microspheres’ diameters, the effect of four easily adjustable processing parameters (hydrogel volume, stirring speed during water in oil emulsion, hydrogel composition and surfactant concentration) on the resulting microspheres diameter was investigated in a full two-level factorial design. The multiple experiments performed indicated that the microsphere diameter was controlled by the stirring speed during water-in-oil emulsion (*p* = 0.0022). The other factors such as the hydrogel volume, hydrogel composition and surfactant concentration did not have any significant effect on the microsphere diameters (*p* = 0.33, 0.28 and 0.11, respectively). The mathematical model obtained from the data correlated well with the experimental results (r^2^ = 81%). Within the factorial design range, the average diameter could be adjusted between 2 and 900 µm in a reproducible manner. Overall, increasing the stirring velocity led to smaller microspheres (Figure 4).

### 3.2. Poly(L-Lysine) Dendrigrafts (DGL)/PEG-Based Hydrogel Is Not Toxic

**Figure 5:**
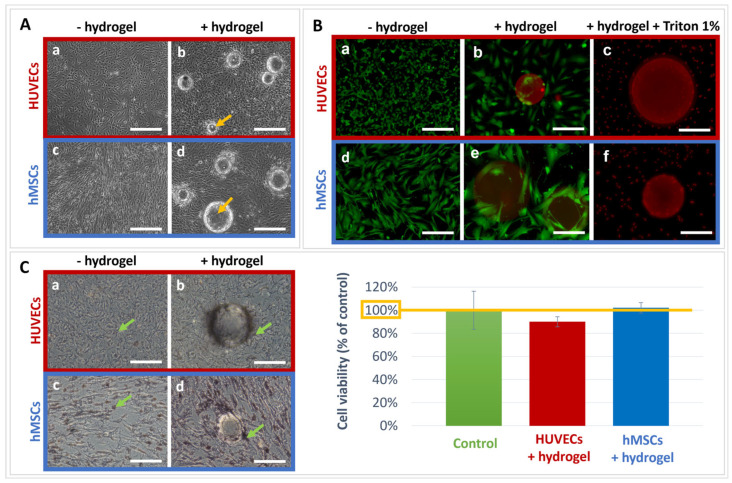
Cell viability. (**A**) Representative images of HUVECs and hMSCs cells cultured without (a,c) or with (b,d) the hydrogel microspheres (yellow arrows) for 72 h under phase-contrast microscopy. Scale bar = 400 µm. (**B**) Representative images of the HUVECs and hMSCs cells labelled with fluorescent Live/Dead^®^ staining (living cells in green; dead cells in red) after culture without (a,d), or with the microspheres (b,e) and the positive control where the cells were incubated with Triton™ 1% (c,f). (a,d) Scale bar = 400 µm; (b,c,e,f) scale bar = 200 µm. (**C**) Representative images of HUVECs and hMSCs cultured with the hydrogel microspheres for 72 h under phase-contrast microscopy (a–d) with visible MTT crystals (green arrows). Scale bar = 200 µm. Cell viability quantification showed no effect of the hydrogel on HUVECs or hMSCs viability. The results are expressed as percentage of control (culture media without hydrogel).

HUVECs and hMSCs monolayers cultured with the hydrogel for 72 h displayed a confluent aspect surrounding the hydrogel microspheres under phase contrast microscopy. The spheres appeared entrapped by the cells which grew around and adhered on the microspheres (Figure 5A). Live/Dead^®^ staining revealed that the cells in contact with the biomaterial were alive as demonstrated by the green fluorescence (Figure 5B). The cell viability was quantified by the MTT test, and the results demonstrated that the microspheres did not affect the HUVECs and the hMSCs viability. The obtained results were comparable to the controls (without hydrogel), indicating an absence of hydrogel toxicity (Figure 5C).

### 3.3. Endothelial Cells Express Complement C5 Gene

**Figure 6:**
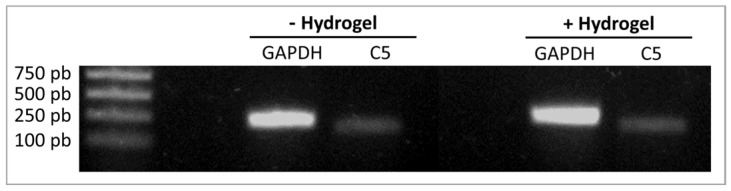
Complement C5 expression by endothelial cells. RT-PCR analysis of C5 mRNA expression shows that HUVECs express C5 mRNA whether incubated with the hydrogel or not. GAPDH was used as a housekeeping control.

RT-PCR analysis revealed that HUVECs express C5 mRNA. This expression did not change after their incubation with the hydrogel (Figure 6).

### 3.4. Poly(L-Lysine) Dendrigrafts (DGL)/PEG-Based Hydrogel Induces TGF-β1 and C5a Secretion from HUVECs

**Figure 7:**
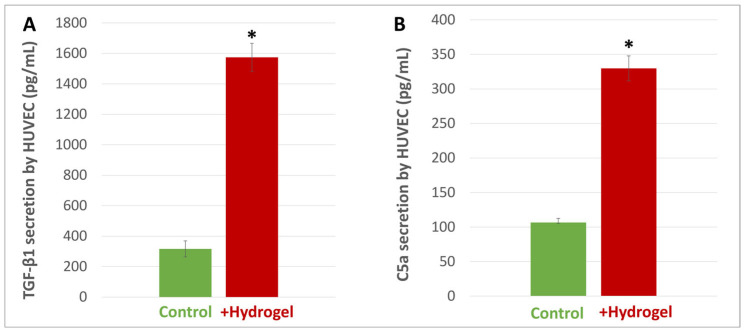
TGF-β1 (**A**) and C5a secretion (**B**) by endothelial cells. After incubating HUVEC cells with the hydrogel for 72 h, enzyme-linked immunosorbent assay showed a significant increase in TGF-β1 and C5a secretion as compared to the controls. Results are expressed in pg/mL. * indicates a statistical difference with the control (*p* < 0.05).

When HUVECs were incubated with the hydrogel, a significant increase in TGF-β1 secretion was observed in the supernatants after 72 h. This secretion was significantly higher with the hydrogel (1600 pg/mL) as compared to the control (300 pg/mL) (Figure 7A). The measurement of C5a in the same supernatants also showed a significant increase after the same delay of 72 h. This secretion was also much higher than the control (respectively, 325 pg/mL versus 100 pg/mL) (Figure 7B).

### 3.5. Incubating HUVECs with Poly(L-Lysine) Dendrigrafts (DGL)/PEG-Based Hydrogel Enhances Mesenchymal Stem Cell Migration

**Figure 8:**
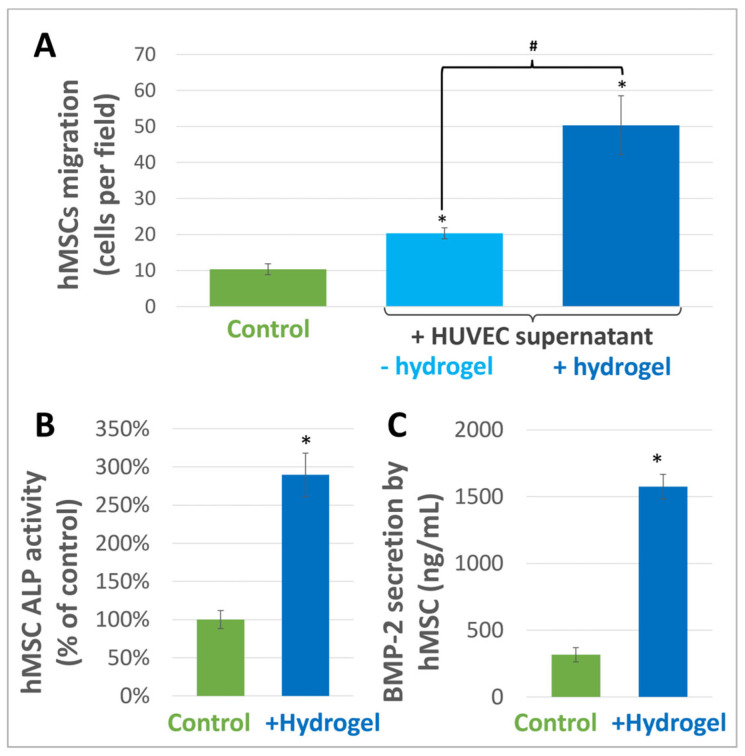
(**A**) Effect of the hydrogel on hMSC migration. Migration of hMSCs significantly increased when they were incubated with HUVEC supernatants in Boyden chambers for 24 h. The increase was significant whether HUVECs were incubated with or without the hydrogel as compared to the control. However, this increase was significantly higher with the hydrogel. Results are expressed as number of cells per field. * indicates a significant difference with the control. # indicates a statistical difference between the two stimulation conditions (+/− hydrogel) (*p* < 0.05). (**B**) hMSC osteogenic differentiation. Incubating hMSCs with the hydrogel for 7 days significantly increased their alkaline phosphatase activity. Results are expressed in percentage of the control. * indicates a statistical difference with the control (*p* < 0.05). (**C**) BMP-2 secretion by hMSCs. After incubating hMSCs with the hydrogel for 72 h, a significant increase in BMP-2 secretion was observed as compared to the control. Results are expressed in ng/mL. * indicates a statistical difference with the control (*p* < 0.05).

When hMSCs were subjected to HUVEC supernatants, they migrated from the upper to the lower compartment of Boyden chambers. This migration was higher than with the control both with and without the hydrogel. However, the increase with the hydrogel was 2.5 times higher than that obtained without the hydrogel (Figure 8A).

### 3.6. Poly(L-Lysine) Dendrigrafts (DGL)/PEG-Based Hydrogel Induces Mesenchymal Stem Cell Osteogenic Differentiation

When hMSCs were incubated with the hydrogel for 7 days, a significant increase in their ALP activity was observed. The ALP activity level was almost three times higher than that of the control (Figure 8B). Measurement of BMP-2 secretion from hMSCs also showed a significant increase after 72 h, reaching 5 times that of the control (Figure 8C).

## 4. Discussion

In this work, we prepared a poly(L-lysine) dendrigrafts (DGL)/PEG-based hydrogel in the form of tunable microspheres to provide an inversed porosity which can be used as a suitable scaffold for bone regeneration.

Numerous fabrication procedures have been used to prepare polymer-based microspheres. These include the emulsion solvent evaporation, spray drying, electro-spinning, gelation followed by emulsification, suspension polymerization, ultrasonication and phase separation [32]. Here, we prepared the hydrogel under the form of microspheres using the water-in-oil emulsion method. This method allows the crosslinking of hydrogel droplets precursor solutions in oil to form the microspheres [33]. This microsphere-type scaffold can provide more versatile applications than pre-shaped scaffolds as it can be directly deposited into various shaped bone defects with only minimally invasive surgery and better recovery [34]. Our fabrication method allowed to prepare spherical microspheres with a diameter that can be controlled by adjusting the stirring speed during the w/o emulsion to form a microsphere-type scaffold, providing a porosity between the microspheres. As demonstrated, the volume of hydrogel, the surfactant concentration or the hydrogel composition had no effect on the microsphere’s synthesis. However, a previous study demonstrated that the mechanical properties of the hydrogel can be tailored by varying the concentrations of each hydrogel components [20]. Here, we demonstrated that this new PEG-based hydrogel cross-linked with DGL is a promising candidate for bone tissue engineering. Several additive manufacturing processes are used in tissue engineering. These allow fabricating the scaffold with the required shape/organization and inner porosity (reviewed in [35]). The objective here is to prepare microspheres of different diameters, mixing different sizes together in order to obtain the inversed porosity which corresponds to the empty spaces left between the microspheres. The function of the hydrogel then would be to create this inversed porosity between the microspheres once injected into the bone defect. Thus, in the initial phase, the hydrogel fills in the defect and maintains the space required for cell recruitment and new blood vessel growth. At a later stage, it will be degraded and replaced by the newly formed bone. Indeed, PEG hydrogel degradation has been studied in vivo by subcutaneous implantation in mice with macrophages. These macrophages were able to degrade the hydrogel after 3 weeks through phagocytosis as demonstrated with histological analysis [30]. In addition, a previous study demonstrated that PEG-based hydrogels inhibit the growth of bacteria in vitro [36]. Both the degradation and antibacterial potential represent important properties of hydrogels in tissue engineering.

The hydrogel biocompatibility was evaluated on two cell types that play pivotal roles in bone regeneration, namely endothelial and mesenchymal stem cells. The hydrogel was biocompatible and did not induce any toxicity as demonstrated with the MTT test. Additionally, the cells adhered on and even entrapped the hydrogel microspheres. Labelling these cells with Live/Dead^®^ Viability/Cytotoxicity kit demonstrated that they were all labelled with the green fluorescence, indicating their viability. The absence of toxicity in the hydrogel in this study is comparable to polycaprolactone/chitosan nanofibers scaffold containing antibacterial agents and ZnO nanoparticles which has been recently developed for use in wound dressing. While the material inhibited bacterial growth, it did not have a significant effect on the viability of L929 Fibroblasts cultured for 24 h directly on the nanofibers, as demonstrated with MTT test [37]. This result is in line with a previous work using the same hydrogel but in a different form where porous hydrogels were prepared by the particulate/leaching technique using paraffin to create the inner porosity. Upon subcutaneous implantation in mice for 3 weeks, the hydrogel was biocompatible and cell infiltration and blood vessel invasion was obtained. This demonstrates the potential of this novel biomaterial for tissue regeneration through the presence of multiple amine groups [20]. Indeed, poly(L-lysine) dendrigrafts (DGL) have numerous advantages for the development of bioengineering materials. They are biocompatible and allow cellular adhesion which adds a bioactive property to the otherwise bio-inert material [31,38]. When DGL was used for surface coating, it has been shown to increase cell adhesion and proliferation [27].

In this study, incubating hMSCs with the hydrogel enhanced their alkaline phosphatase activity, which is considered as an early marker of osteogenic differentiation. This activity strongly increased after 7 days of cell culture with the hydrogel. Although we did not investigate the mechanism of this increase, our result is in agreement with previously published data which reported ALP activity being highest between 4 and 7 days of cell culture [39]. This result is confirmed by the quantification of BMP-2 growth factor secretion as our study showed that the hydrogel enhanced BMP-2 secretion by hMSCs. This growth factor is known to promote bone formation by directing hMSC differentiation into osteoblasts/osteocytes [40,41]. In ectopic bone formation, BMP-2 plays an important role in the rapid induction of bone matrix by remodeling mature bone similar to that observed in normal bone development [40]. The osteogenic potential of the hydrogel reported in our study is similar to those obtained with a gelatin-based nanocomposite scaffold developed by loading zoledronic acid molecules. These represent a subset of synthetic small molecules used as the main drugs to stimulate the growth and differentiation of osteoblastic cells, increasing bone formation and preventing bone loss. When human adipose stem cells were seeded on the prepared scaffolds, zoledronic acid increased the cell proliferation, showed a high viability rate as obtained with the MTT assay. This was associated with an increased osteogenic differentiation as demonstrated with an increase in alkaline phosphatase level and mineralization [42]. Our data are also in line with another study using nano hydroxyapatite/collagen scaffold to encapsulate Narigirin, a natural flavonoid. This scaffold increased alkaline phosphatase activity, the formation of calcium nodules, and a higher expression of osteogenic-related genes such as Osteocalcin, BMP-2, and Osteopontin. When administered into the rats with skull defects, the scaffold significantly promoted the reconstruction of bone tissues and the early repair of skull defects [43].

Taken together, both ALP activity and BMP-2 secretion suggest that the developed hydrogel induced hMSC osteogenic differentiation. However, the mechanism beyond this induction needs to be investigated.

An original aspect of this work is the consequence of the hydrogel interaction with endothelial cells which occurs upon placement of the scaffold in vivo. In agreement with previously published studies [44], our work shows that endothelial cells express Complement C5. However, a novelty of our study is the demonstration that the addition of the hydrogel lead to a significant increase of C5a release from endothelial cells which, to our knowledge, has never been reported.

Complement proteins are produced by the liver and some immune cells [45,46]. They are known for their well-established roles during the inflammation process where their efficiency in eliminating pathogens has been well studied and demonstrated [47]. Interestingly, expression of complement receptors on cell types other than the inflammatory cells suggested Complement implication in other processes such as tissue regeneration as recently demonstrated in dental pulp mesenchymal stem cells [48].

Investigating the relationships between Complement and bone regeneration reported the expression of C5aR in mesenchymal stem cells as well as in osteoblasts [49]. This expression appears to be modulated during the regeneration process. Indeed, when C5aR expression pattern was investigated after tibia fracture in rats, C5aR was expressed by osteoblasts from 3 up to 28 days in the newly formed bone [50], indicating its implication in the bone regeneration process.

This implication has further been shown recently in vitro by demonstrating that Complement C5a plays a significant role in BMMSCs recruitment. Indeed, when Complement C5a produced by injured periodontal ligament cells (PDL) was incubated with BMMSCs, C5a bound to their C5aR and induced its subsequent phosphorylation leading to their proliferation and recruitment towards injured PDL cells. When bone filling materials were applied onto the injured PDL cells, they modulated C5a production. Indeed, C5a secretion by injured PDLs level doubled, its binding to BMMSC C5aR significantly increased, leading to an increased receptor phosphorylation and subsequent increase in stem cell proliferation and recruitment to the materials’ application site [51].

While the mechanism by which C5a release upon cell interaction with a given material remains to be elucidated, our work demonstrates Complement C5a implication in two important steps for bone regeneration: mesenchymal stem cell proliferation and recruitment to the stimulation/injury site.

Thus, the release of C5a when endothelial cells are incubated with the hydrogel strongly suggests that endothelial cells represent a major actor of bone regeneration by providing a C5a gradient for hMSCs recruitment.

Interestingly, interaction of the hydrogel with the HUVECs also induced the secretion of TGF-β1. This growth factor is ubiquitous in skeletal tissue playing major roles in maintenance of bone metabolism through the control of cellular proliferation, differentiation, and migration [52]. It is stored in a latent form in the bone, and it is activated upon bone injury/fracture. During bone resorption, TGF-β1 release by osteoclasts generates a gradient that induces hMSCs recruitment to the bone surface [53]. Similarly, the increase in TGF-β1 obtained after incubating HUVECs with the hydrogel and the subsequent mesenchymal stem cell recruitment obtained here is in line with these findings.

## 5. Conclusions

Overall, this work allowed to develop a poly(L-lysine) dendrigrafts (DGL) hydrogel in the form of microspheres with tunable diameters varying from 10 to 1600 µm to provide an inversed porosity. Its interaction with endothelial cells increased the secretion of bioactive molecules such as C5a by 3-fold and TGF-β1 by 5-fold, and enhanced mesenchymal stem cell recruitment by 2.5-fold. Furthermore, the hydrogel interaction with these cells enhanced their osteogenic potential.

Even if our study did not provide an explanation for the possible mechanisms of the hydrogel osteogenic potential, these results appear promising and deserves further investigations for the future applications of the hydrogel in tissue engineering.

Within the limit of this in vitro study, this poly(L-lysine) dendrigrafts (DGL) hydrogel appears promising for bone regeneration.

## Data Availability

Not applicable.

## References

[1] Loi F., Córdova L.A., Pajarinen J., Lin T., Yao Z., Goodman S.B. (2016). Inflammation, Fracture and Bone Repair. Bone.

[2] Dimitriou R., Jones E., McGonagle D., Giannoudis P.V. (2011). Bone Regeneration: Current Concepts and Future Directions. BMC Med..

[3] Zhu G., Zhang T., Chen M., Yao K., Huang X., Zhang B., Li Y., Liu J., Wang Y., Zhao Z. (2021). Bone Physiological Microenvironment and Healing Mechanism: Basis for Future Bone-Tissue Engineering Scaffolds. Bioact. Mater..

[4] Ho-Shui-Ling A., Bolander J., Rustom L.E., Johnson A.W., Luyten F.P., Picart C. (2018). Bone Regeneration Strategies: Engineered Scaffolds, Bioactive Molecules and Stem Cells Current Stage and Future Perspectives. Biomaterials.

[5] Colnot C. (2009). Skeletal Cell Fate Decisions Within Periosteum and Bone Marrow During Bone Regeneration. J. Bone Miner. Res..

[6] Roberts T.T., Rosenbaum A.J. (2012). Bone Grafts, Bone Substitutes and Orthobiologics. Organogenesis.

[7] Wang W., Yeung K.W.K. (2017). Bone Grafts and Biomaterials Substitutes for Bone Defect Repair: A Review. Bioact. Mater..

[8] Gibbs D.M.R., Black C.R.M., Dawson J.I., Oreffo R.O.C. (2016). A Review of Hydrogel Use in Fracture Healing and Bone Regeneration. J. Tissue Eng. Regen. Med..

[9] Giannoudis P.V., Dinopoulos H., Tsiridis E. (2005). Bone Substitutes: An Update. Injury.

[10] Robinson P.G., Abrams G.D., Sherman S.L., Safran M.R., Murray I.R. (2020). Autologous Bone Grafting. Oper. Tech. Sport. Med..

[11] Spicer C.D. (2020). Hydrogel Scaffolds for Tissue Engineering: The Importance of Polymer Choice. Polym. Chem..

[12] Sohn H.-S., Oh J.-K. (2019). Review of Bone Graft and Bone Substitutes with an Emphasis on Fracture Surgeries. Biomater. Res..

[13] Dreifke M.B., Ebraheim N.A., Jayasuriya A.C. (2013). Investigation of Potential Injectable Polymeric Biomaterials for Bone Regeneration. J. Biomed. Mater. Res. Part A.

[14] Hunt J.A., Chen R., van Veen T., Bryan N. (2014). Hydrogels for Tissue Engineering and Regenerative Medicine. J. Mater. Chem. B.

[15] Ma Z., Gao C., Gong Y., Shen J. (2003). Paraffin Spheres as Porogen to Fabricate Poly(L-Lactic Acid) Scaffolds with Improved Cytocompatibility for Cartilage Tissue Engineering. J. Biomed. Mater. Res. B Appl. Biomater..

[16] Zaborowska M., Bodin A., Bäckdahl H., Popp J., Goldstein A., Gatenholm P. (2010). Microporous Bacterial Cellulose as a Potential Scaffold for Bone Regeneration. Acta Biomater..

[17] de Kruijff B., Cullis P.R. (1980). The Influence of Poly(L-Lysine) on Phospholipid Polymorphism. Evidence That Electrostatic Polypeptide-Phospholipid Interactions Can Modulate Bilayer/Non-Bilayer Transitions. Biochim. Biophys. Acta.

[18] Huang R., Liu S., Shao K., Han L., Ke W., Liu Y., Li J., Huang S., Jiang C. (2010). Evaluation and Mechanism Studies of PEGylated Dendrigraft Poly-L-Lysines as Novel Gene Delivery Vectors. Nanotechnology.

[19] Chinnadayyala S.R., Park J., Choi Y., Han J.-H., Yagati A.K., Cho S. (2019). Electrochemical Impedance Characterization of Cell Growth on Reduced Graphene Oxide–Gold Nanoparticles Electrodeposited on Indium Tin Oxide Electrodes. Appl. Sci..

[20] Carrancá M., Griveau L., Remoué N., Lorion C., Weiss P., Orea V., Sigaudo-Roussel D., Faye C., Ferri-Angulo D., Debret R. (2020). Versatile Lysine Dendrigrafts and Polyethylene Glycol Hydrogels with Inherent Biological Properties: In Vitro Cell Behavior Modulation and in Vivo Biocompatibility. J. Biomed. Mater. Res..

[21] Li X., Ji J., Pu M., Wang X., Shen J. (2008). Surface Tailoring of Poly(Ethylene Terephthalate) via Ligand-Tethered Comb-like PEG to Enhance Endothelialization. J. Mater. Sci: Mater. Med..

[22] Wang G.-X., Deng X.-Y., Tang C.-J., Liu L.-S., Xiao L., Xiang L.-H., Quan X.-J., Legrand A.P., Guidoin R. (2006). The Adhesive Properties of Endothelial Cells on Endovascular Stent Coated by Substrates of Poly-L-Lysine and Fibronectin. Artif. Cells Blood Substit. Biotechnol..

[23] Lu H., Guo L., Kawazoe N., Tateishi T., Chen G. (2009). Effects of Poly(L-Lysine), Poly(Acrylic Acid) and Poly(Ethylene Glycol) on the Adhesion, Proliferation and Chondrogenic Differentiation of Human Mesenchymal Stem Cells. J. Biomater. Sci. Polym. Ed..

[24] Tian B., Wang N., Jiang Q., Tian L., Hu L., Zhang Z. (2021). The Immunogenic Reaction and Bone Defect Repair Function of ε-Poly-L-Lysine (EPL)-Coated Nanoscale PCL/HA Scaffold in Rabbit Calvarial Bone Defect. J. Mater. Sci. Mater. Med..

[25] Kim Y.S., Chien A.J., Guo J.L., Smith B.T., Watson E., Pearce H.A., Koons G.L., Navara A.M., Lam J., Scott D.W. (2020). Chondrogenesis of Cocultures of Mesenchymal Stem Cells and Articular Chondrocytes in Poly(l-Lysine)-Loaded Hydrogels. J. Control. Release.

[26] Heo J.S., Kim H.O., Song S.Y., Lew D.H., Choi Y., Kim S. (2016). Poly-L-Lysine Prevents Senescence and Augments Growth in Culturing Mesenchymal Stem Cells Ex Vivo. Biomed. Res. Int..

[27] Lorion C., Faye C., Maret B., Trimaille T., Régnier T., Sommer P., Debret R. (2014). Biosynthetic Support Based on Dendritic Poly(L-Lysine) Improves Human Skin Fibroblasts Attachment. J. Biomater. Sci. Polym. Ed..

[28] Zhu C., Hard C., Lin C., Gitsov I. (2005). Novel Materials for Bioanalytical and Biomedical Applications: Environmental Response and Binding/Release Capabilities of Amphiphilic Hydrogels with Shape-Persistent Dendritic Junctions. J. Polym. Sci. Part A Polym. Chem..

[29] Grinstaff M.W. (2008). Dendritic Macromers for Hydrogel Formation: Tailored Materials for Ophthalmic, Orthopedic, and Biotech Applications. J. Polym. Sci. Part A Polym. Chem..

[30] Griveau L., Lafont M., le Goff H., Drouglazet C., Robbiani B., Berthier A., Sigaudo-Roussel D., Latif N., Visage C.L., Gache V. (2021). Design and Characterization of an in Vivo Injectable Hydrogel with Effervescently Generated Porosity for Regenerative Medicine Applications. Acta Biomater..

[31] Francoia J.-P., Vial L. (2018). Everything You Always Wanted to Know about Poly-L-Lysine Dendrigrafts (But Were Afraid to Ask). Chem. Eur. J..

[32] Hossain K.M.Z., Patel U., Ahmed I. (2015). Development of Microspheres for Biomedical Applications: A Review. Prog. Biomater..

[33] Zhao Z., Wang Z., Li G., Cai Z., Wu J., Wang L., Deng L., Cai M., Cui W. (2021). Injectable Microfluidic Hydrogel Microspheres for Cell and Drug Delivery. Adv. Funct. Mater..

[34] Hu X., Shen H., Yang F., Liang X., Wang S., Wu D. (2014). Modified Composite Microspheres of Hydroxyapatite and Poly(Lactide-Co-Glycolide) as an Injectable Scaffold. Appl. Surf. Sci..

[35] Wu Y., Lu Y., Zhao M., Bosiakov S., Li L. (2022). A Critical Review of Additive Manufacturing Techniques and Associated Biomaterials Used in Bone Tissue Engineering. Polymers.

[36] Liu S., Jiang T., Guo R., Li C., Lu C., Yang G., Nie J., Wang F., Yang X., Chen Z. (2021). Injectable and Degradable PEG Hydrogel with Antibacterial Performance for Promoting Wound Healing. ACS Appl. Bio Mater..

[37] Mosallanezhad P., Nazockdast H., Ahmadi Z., Rostami A. (2022). Fabrication and Characterization of Polycaprolactone/Chitosan Nanofibers Containing Antibacterial Agents of Curcumin and ZnO Nanoparticles for Use as Wound Dressing. Front. Bioeng. Biotechnol..

[38] Yuan Y., Shi X., Gan Z., Wang F. (2018). Modification of Porous PLGA Microspheres by Poly-l-Lysine for Use as Tissue Engineering Scaffolds. Colloids Surf. B Biointerfaces.

[39] Mauney J.R., Blumberg J., Pirun M., Volloch V., Vunjak-Novakovic G., Kaplan D.L. (2004). Osteogenic Differentiation of Human Bone Marrow Stromal Cells on Partially Demineralized Bone Scaffolds in Vitro. Tissue Eng..

[40] Lieberman J.R., Daluiski A., Einhorn T.A. (2002). The Role of Growth Factors in the Repair of Bone. Biology and Clinical Applications. J. Bone Joint. Surg. Am..

[41] Bessa P.C., Casal M., Reis R.L. (2008). Bone Morphogenetic Proteins in Tissue Engineering: The Road from the Laboratory to the Clinic, Part I (Basic Concepts). J. Tissue Eng. Regen. Med..

[42] Abdulahy S.B., Esmaeili Bidhendi M., Vaezi M.R., Moosazadeh Moghaddam M. (2022). Osteogenesis Improvement of Gelatin-Based Nanocomposite Scaffold by Loading Zoledronic Acid. Front. Bioeng. Biotechnol..

[43] Zuo Y., Li Q., Xiong Q., Li J., Tang C., Zhang Y., Wang D. (2022). Naringin Release from a Nano-Hydroxyapatite/Collagen Scaffold Promotes Osteogenesis and Bone Tissue Reconstruction. Polymers.

[44] Sartain S.E., Turner N.A., Moake J.L. (2016). TNF Regulates Essential Alternative Complement Pathway Components and Impairs Activation of Protein C in Human Glomerular Endothelial Cells. J. Immunol..

[45] Morris K.M., Aden D.P., Knowles B.B., Colten H.R. (1982). Complement Biosynthesis by the Human Hepatoma-Derived Cell Line HepG2. J. Clin. Investig..

[46] Lubbers R., van Essen M.F., van Kooten C., Trouw L.A. (2017). Production of Complement Components by Cells of the Immune System. Clin. Exp. Immunol..

[47] Ehrnthaller C., Ignatius A., Gebhard F., Huber-Lang M. (2011). New Insights of an Old Defense System: Structure, Function, and Clinical Relevance of the Complement System. Mol. Med..

[48] Chmilewsky F., Jeanneau C., Laurent P., About I. (2014). Pulp Fibroblasts Synthesize Functional Complement Proteins Involved in Initiating Dentin-Pulp Regeneration. Am. J. Pathol..

[49] Ignatius A., Schoengraf P., Kreja L., Liedert A., Recknagel S., Kandert S., Brenner R.E., Schneider M., Lambris J.D., Huber-Lang M. (2011). Complement C3a and C5a Modulate Osteoclast Formation and Inflammatory Response of Osteoblasts in Synergism with IL-1β. J. Cell. Biochem..

[50] Ignatius A., Ehrnthaller C., Brenner R.E., Kreja L., Schoengraf P., Lisson P., Blakytny R., Recknagel S., Claes L., Gebhard F. (2011). The Anaphylatoxin Receptor C5aR Is Present during Fracture Healing in Rats and Mediates Osteoblast Migration in Vitro. J. Trauma.

[51] Jeanneau C., Le Fournis C., About I. (2019). Xenogeneic Bone Filling Materials Modulate Mesenchymal Stem Cell Recruitment: Role of the Complement C5a. Clin. Oral Investig..

[52] Janssens K., ten Dijke P., Janssens S., Van Hul W. (2005). Transforming Growth Factor-Β1 to the Bone. Endocr. Rev..

[53] Tang Y., Wu X., Lei W., Pang L., Wan C., Shi Z., Zhao L., Nagy T.R., Peng X., Hu J. (2009). TGF-Beta1-Induced Migration of Bone Mesenchymal Stem Cells Couples Bone Resorption with Formation. Nat. Med..

